# Replication characteristics of swine influenza viruses in precision-cut lung slices reflect the virulence properties of the viruses

**DOI:** 10.1186/1297-9716-44-110

**Published:** 2013-11-13

**Authors:** Fandan Meng, Darsaniya Punyadarsaniya, Sabine Uhlenbruck, Isabel Hennig-Pauka, Christel Schwegmann-Wessels, Xiaofeng Ren, Ralf Dürrwald, Georg Herrler

**Affiliations:** 1 Institute of Virology University of Veterinary Medicine, Hannover, Germany; 2 Immunology and Virology Department, Faculty of Veterinary Medicine, Mahanakorn University of Technology, Bangkok, Thailand; 3 Clinic for Swine and Small Ruminants, University of Veterinary Medicine, Hannover, Germany; 4 College of Veterinary Medicine, Northeast Agricultural University, Harbin, China; 5 IDT Biologika GmbH, Dessau-Roβlau, Germany; 6 Present address: Clinic for Swine, University of Veterinary Medicine, Vienna, Austria

## Abstract

Precision-cut lung slices of pigs were infected with five swine influenza A viruses of different subtypes (A/sw/Potsdam/15/1981 H1N1, A/sw/Bad Griesbach/IDT5604/2006 H1N1, A/sw/Bakum/1832/2000 H1N2, A/sw/Damme/IDT5673/2006 H3N2, A/sw/Herford/IDT5932/2007 H3N2). The viruses were able to infect ciliated and mucus-producing cells. The infection of well-differentiated respiratory epithelial cells by swine influenza A viruses was analyzed with respect to the kinetics of virus release into the supernatant. The highest titres were determined for H3N2/2006 and H3N2/2007 viruses. H1N1/1981 and H1N2/2000 viruses replicated somewhat slower than the H3N2 viruses whereas a H1N1 strain from 2006 multiplied at significantly lower titres than the other strains. Regarding their ability to induce a ciliostatic effect, the two H3N2 strains were found to be most virulent. H1N1/1981 and H1N2/2000 were somewhat less virulent with respect to their effect on ciliary activity. The lowest ciliostatic effect was observed with H1N1/2006. In order to investigate whether this finding is associated with a corresponding virulence in the host, pigs were infected experimentally with H3N2/2006, H1N2/2000, H1N1/1981 and H1N1/2006 viruses. The H1N1/2006 virus was significantly less virulent than the other viruses in pigs which was in agreement with the results obtained by the in vitro-studies. These findings offer the possibility to develop an ex vivo-system that is able to assess virulence of swine influenza A viruses.

## Introduction

Influenza A viruses are a major cause of acute respiratory disease in pigs. Typical disease is characterized by high fever, loss of appetite, depression, tachypnoea, abdominal breathing and, less frequently, coughing. Mortality rates are low, but morbidity rates can be as high as 100% [1]. Serological surveys suggest that there are many infections that do not result in acute disease as indicated by the high number of positive reactions especially against H1N1 viruses [2].

Swine influenza virus (swFLUAV) strains that are endemic in swine populations worldwide are assigned to the subtypes H1N1, H3N2, or H1N2. Depending on the geographic distribution, these viruses differ in their origin as well as in their genetic and antigenic properties [1,3]. H1N1 swFLUAVs prevalent in Europe are entirely of avian origin and were introduced into the swine population in 1979 [4]. This swFLUAV lineage is designated “avian-like”. H3N2 swFLUAVs became widespread in European pigs in the 1980s [5]. These viruses have maintained the genomic RNA segments coding for the internal and non-structural proteins from the “avian-like” H1N1 swFLUAV, acquired the RNA segments coding for the haemagglutinin (HA) and the neuraminidase (NA) from descendants of the human pandemic A/Hong Kong/1/68 (H3N2) virus [6,7]. The genotype of these reassortant H3N2 viruses is retained in the H1N2 viruses except for the HA segment which has been acquired from a human H1N1 virus of the 1980s [8-11] and a slightly modified NA segment which reflects drift events in the NA of H3N2 human influenza A viruses [12]. Seroprevalence studies indicate that the H1N1, H3N2 and H1N2 swFLUAVs co-circulate in swine populations [2,13-15]. Additional reassortants with different HA/NA gene combinations may occur but are not predominant, e.g. H1N2 viruses with “avian-like” HA from H1N1 swFLUAVs [9,10,16,17].

Primary target cells for influenza viruses are cells of the respiratory epithelium [18]. In vitro studies with differentiated respiratory epithelial cells are possible, e.g. by using air-liquid interface cultures or explant cultures. The former culture system has been used to analyze the infection by human influenza viruses [19,20]. In the case of differentiated airway epithelial cells from pigs, infection studies with influenza viruses have been reported with explant cultures either from the trachea [21] or from different parts for the respiratory tract [22]. We have recently reported that precision-cut lung slices (PCLS) are a valuable culture system for porcine differentiated respiratory epithelial cells. This culture system has been used for various scientific fields, but rarely for infection studies [23,24]. Interesting features of PCLS are that (i) they can be obtained in large numbers, (ii) differentiated epithelial cells are maintained in their original setting, and (iii) they are viable for more than a week. Recently, the infection of PCLS has been reported to characterize an A/sw/Bissendorf/IDT1864/2003 H3N2 swFLUAV [25]. Here we used this culture system to compare the infection of respiratory epithelial cells by five swine influenza A viruses derived from the three subtypes, H1N1, H1N2, and H3N2. Replication properties of these viruses in porcine airway epithelial cells were found to reflect the virulent properties determined in corresponding animal experiments.

## Materials and methods

### Cells and viruses

MDKC II, a subline of Madin-Darby canine kidney cells [26] were maintained in Eagle’s minimal essential medium (EMEM) supplemented with 10% fetal calf serum (Biochrom AG, Berlin). The cells were incubated in a humidified atmosphere containing 5% CO_2_ at 37 °C and passaged every 2–3 days.

Swine influenza viruses of the H1N1 subtype (A/sw/Potsdam/15/1981) H1N1/1981, the H1N2 subtype (A/sw/Bakum/1832/2000) H1N2/2000 and the H3N2 subtype (A/sw/Herford/IDT5932/2007) H3N2/2007 were provided by Prof. Michaela Schmidtke, University of Jena, Germany. The H1N1 subtype (A/sw/Bad Griesbach/IDT5604/2006) H1N1/2006 and the H3N2 subtype (A/sw/Damme/IDT5673/2006) H3N2/2006 were obtained from IDT Biologika GmbH, Dessau-Rosslau, Germany. The strains had been originally isolated from pigs with respiratory disease during surveillance programs initiated by Prof. Jochen Süss, Berlin (strains Potsdam and Bakum) and Dr. Ralf Dürrwald, Dessau-Rosslau (strains with IDT numbering; IDT, Impfstoffwerk Dessau-Tornau, now IDT Biologika GmbH). Virus stocks were propagated by infection of MDCK cells at low multiplicity of infection and incubation in infection medium (Eagle’s minimal essential medium (EMEM)) containing acetylated trypsin 1 μg/mL (Sigma-Aldrich, Munich). Supernatants were clarified by low-speed centrifugation (200 × *g*, 10 min) and stored at -80 °C.

### Precision-cut lung slices

Precision-cut lung slices were prepared from lungs of three months old healthy crossbred pigs which were obtained from conventional farms and housed in the Clinics for Swine and Small Ruminants and Forensic Medicine at the University of Veterinary Medicine, Hannover. The animals had a good health status and no clinical symptoms. The cranial, middle, and intermediate lobes of the fresh lungs were carefully removed and filled with 37 °C warm low-melting agarose (AGAROSE LM; GERBU, Gaiberg, Germany) as previously described [23,25,27]. After the agarose became solidified on ice, the tissue was stamped out as cylindrical portions (8-mm tissue coring tool) and approx. 250 μm thick slices were prepared by using the Krumdieck tissue slicer (TSE systems, model MD4000-01) with a cycle speed of 60 slices/min [25]. PCLS were incubated in 1 mL of RPMI 1640 medium (Invitrogen/Gibco, Germany) containing antibiotics and antimycotics (2.5 mg Amphotericin B/L, 1 mg Clotrimazole/L, 10 mg Enrofloxacin/L, 50 mg Kanamycin/L, 1:100 dilution of Penicillin/Streptomycin stock solution containing 10 000 U Penicillin G/mL and 10 mg Streptomycin/mL) per slice in 24-well plate at 37 °C and 5% CO_2_. In order to remove the agarose, culture medium was changed every hour during the first four hours and once per day for further culture. These extensive washings may have also removed preexisting antibodies - if present -, because no neutralizing activity was ever observed.

The viability was analyzed by observing the ciliary activity under the light microscope (Zeiss Axiovert 35) equipped with an ORCA C4742-80 digital camera (Hamamatsu) and SIMPLE-PCI analysis software (Compix Imaging Systems). In selected samples, the slices were analyzed for bronchoconstriction by addition of 10^-4^ M methacholine (acetyl-β-methylcholine chloride, Sigma Aldrich) as described previously [23,25]. The integrity of the cells was also determined by applying a Live/Dead viability/cytotoxicity assay kit (Fluo Probes, FPBE4710). For this purpose, the slices were washed with phosphate-buffered saline (PBS) and incubated with Calcein AM (1 μM) and ethidium bromide (EthD-1; 2 μM) for 30 min. After incubation, slices were washed with PBS, embedded in Mowiol resin and analyzed using a Leica TCS SP5 AOBS confocal laser scanning microscope.

### Virus infection and titration

For infection experiments, three slices were used for each virus and all trials were repeated three times. PCLS were infected by different subtypes of swine influenza viruses (H1N1, H1N2, H3N2) in a volume of 300 μL/slice at 10^5^ TCID_50_/mL (50% tissue culture infectious dose/mL). Supernatants from virus-infected and uninfected control slices were collected at different time points (0, 8, 24, 48, 72, 96, 120, 144, 168 h) post-infection and the samples were stored at -80 °C. For virus infectivity analysis, virus titers were determined by endpoint dilution titration on MDCK cells in 96-well plates as described previously [28]. Briefly, for each sample, 10-fold serial dilution steps were performed. From each dilution, 100 μL/well was added onto confluent MDCK cells in a 96-well plate and every sample had 6 replicates. Plates were incubated for 72 h and the wells were visually analyzed for virus-induced cytopathogenic effects.

### Ciliary activity assay

PCLS were analyzed under a light microscope to estimate the ciliary activity as described previously [25]. Each bronchus was virtually divided into ten segments, each of which was monitored for the presence or absence of ciliary activity. The ciliostatic effect was determined by estimating the percentage of the luminal surface showing ciliary activity, as it is common practice to evaluate ciliary activity of tracheal organ cultures [29]. Slices that showed 100% ciliary activity were selected for further viral infection experiments.

### Cryosections

PCLS were mounted on small filter paper with tissue-freezing medium (Jung, Heidelberg, Germany), frozen in liquid nitrogen and kept in -80 °C prior to cutting. Slices were cut at 10 μm thickness by a cryostat (Reichert-Jung, Nuβloch, Germany). The sections were dried overnight at room temperature and kept frozen at -20 °C until staining.

### Immunofluorescence analysis of cryosections

Sections with a central bronchus were fixed with 3% paraformaldehyde for 20 min followed by 5 min incubation with 0.1 M glycine and three washing steps with PBS. Then, the sections were permeabilized with 0.2% Triton X-100 for 20 min at room temperature followed by 3 washing steps with PBS. All antibodies were diluted in 1% bovine serum albumin and incubated with the sections for 1 h at room temperature in a humid incubation chamber. For detection of virus particles, monoclonal antibody against the influenza A virus nucleoprotein (NP) (AbDSeroTec, Düsseldorf) at a 1:750 dilution was used followed by incubation with anti-mouse IgG (Sigma-Aldrich) secondary antibody. For detection of infected cells, ciliated and mucus-producing cells were stained by antibodies against β-tubulin and mucin, respectively [19,20,23,24]. In brief, the ciliated cells were visualized with a Cy3-labeled monoclonal antibody recognizing β-tubulin (1:500; Sigma-Aldrich) and the mucus-producing cells were stained by mucin-5 AC antibody (1:100; Santa Cruz Biotechnology), followed by an anti-rabbit IgG secondary antibody (Sigma-Aldrich). Nuclei were stained by DAPI (4′,6-diamidino-2-phenylindole). Finally, all the samples were embedded in Mowiol and stored at 4 °C until examination under the confocal microscope.

### Animal experiments

All trial procedures and animal care activities were conducted under approval of Good Clinical Practice (VICH GL9, CVMP/VICH/595/98) and German Animal Protection Law at IDT Biologika GmbH. The protocol IDT A 3/2004 was approved by the Landesverwaltungsamt Sachsen-Anhalt (Reference Number: AZ 42502-3-401 IDT). A total of 48 crossbred swine (Piétrain x Large White; IDT Biologika GmbH, Dessau-Roβlau, Germany) were used in the present study. Pigs had been proved to be free of influenza during their life span as well as free of maternally-derived antibodies against influenza A viruses. They were housed in identical isolation rooms based on their challenge status and were provided with feed and water ad libitum. Experimental infections were done in the BSL-2 infection units of IDT Biologika GmbH, Dessau-Roβlau, Germany, which are equipped with High Efficiency Particulate Airfilter H13 filters. Four independent trials were performed for each of the four viruses examined. The experimental design is summarized in Table 1. In each trial 12 pigs were included. At an age of 11 weeks, all 12 pigs were challenged by one-hour-aerosol exposure with the same virus in each trial. Aerosols were dispersed through a flow aerosol generator which produces droplets of 0.5 to 20 μm under atmospheric pressure. The infections were carried out under high dose infection conditions (> 10^7^ TCID_50_/m^3^).

**Table 1:** Overview of the experimental design of pig infection experiments with influenza A Viruses

**Strain**	**Subtype**	**Abbreviation**	**Number of pigs**
**swFLUAV/…**			Total/lung investigation*
Potsdam/1/1981	Original avH1N1°	H1N1/1981	12/5
Bad Griesbach/IDT5604/2006	Recent avH1N1	H1N1/2006	12/5
Damme/IDT5673/2006	huH3N2	H3N2/2006	12/6
Bakum/1832/2000	huH1N2	H1N2/2000	12/7

*the total number of pigs was available for analysis on 0 and 1 dpi; the second number corresponds to pigs that were killed for lung investigation on 1 dpi; the difference between both numbers reflects the pigs that were observed until day 3 pi; °original means isolated shortly after introduction of avian-like H1N1 (avH1N1) viruses into the Eurpean swine population; hu, human-like.

After infection, rectal temperatures and signs of respiratory disease, dyspnoea, and cough were recorded twice daily 1–3 days post-infection (dpi). Dyspnoea was assessed as follows: 1, increased respiratory frequency and moderate flank movement; 2, marked breathing difficulty and severe flank movement; 3, laboured breathing affecting the entire body, pronounced flank movement and substantial movements of the snout; 4, extreme breathing difficulty reflecting substantial lack of oxygen.

In each trial 5–7 animals were stunned by electrical stunning tongs 1 dpi and bled to death. Lung tissue samples were taken from each lobe for virus detection. Samples of the right and left halves of the lungs were pooled, ground with sterile sea sand, and diluted 1:10 in dilution medium (1.0 mL Amphotericin B and 0.1 mL Gentamycin, made up to 100 mL with phosphate buffered saline solution). The virus load in the lungs was determined by titration of the lung samples in embryonated hens eggs. Dilution series (log_10_) from lung samples were injected into the allantois cavity of 11-day-old chicken embryos (0.1 mL; 5 eggs per dilution). After sealing the perforation point eggs were incubated at 37 °C and checked daily for vitality using an egg candler. On day 4 post-infection (pi), the allantois fluid was collected and tested in the haemagglutination test. The Spearman and Kaerber method was used to calculate the EID_50_ from the haemagglutinating activity.

Mann–Whitney-U-test was performed to evaluate statistical significances for data obtained in the animal experiments by using the program SPSS 15.0.

## Results

**Figure 1:**
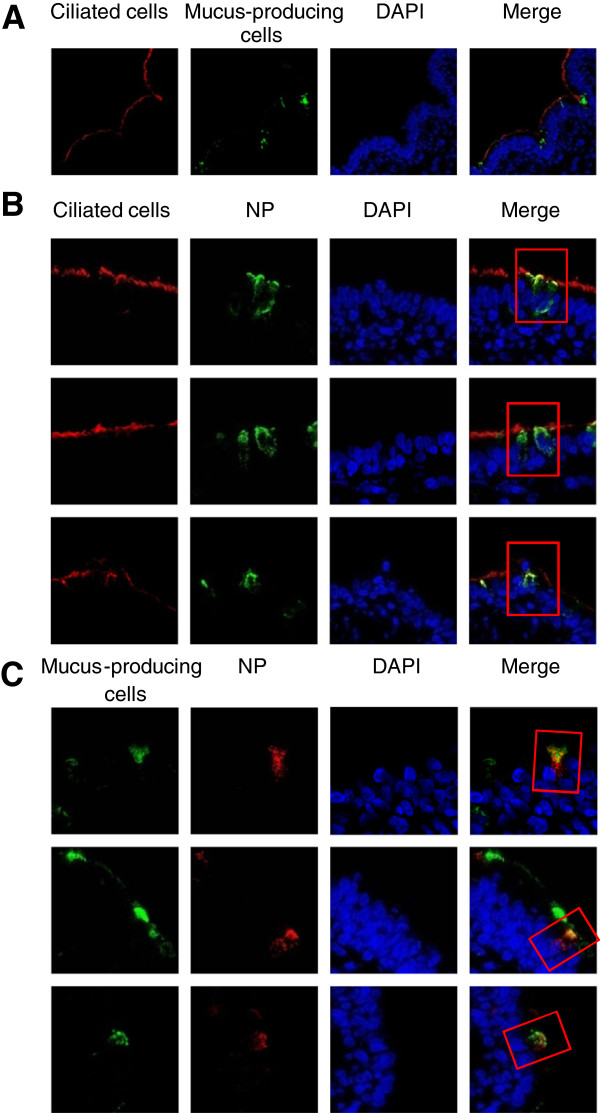
**Immunostaining of swine PCLS infected by swine influenza A viruses.** PCLS were infected by subtypes H1N1 (H1N1/1981), H1N2 (H1N2/2000) and H3N2 (H3N2/2007) swine influenza viruses. Cryosections were prepared at 24 hpi and used for detection of infected cells, ciliated cells, and mucus-producing cells. Infected cells were stained with anti-nucleoprotein (NP) antibody; ciliated cells were stained using anti-β-tubulin antibody (red) and mucus-producing cells were stained using anti-Muc5Ac antibody (green). **A**, control slices showing ciliated and mucus-producing cells; **B**, co-staining of ciliated and virus-infected cells; **C**, co-staining of mucus-producing cells and virus-infected cells. Nuclei were stained with 4′,6-diamidino-2-phenylindole (DAPI).

In a previous study we have characterized the infection of swine PCLS by strain A/Bissendorf/IDT1864/2003 (H3N2) [25] and found that it infected both ciliated cells and mucus-producing cells. Furthermore, we determined the ciliostatic effect and analyzed the kinetics of virus release. Here we extended our investigation to five additional strains comprising all three subtypes prevailing in the swine population, H1N1, H1N2, and H3N2: A/Potsdam/1/1981 (H1N1) H1N1/1981, A/Bad Griesbach/IDT5604/2006 (H1N1) H1N1/2006, A/Bakum/1832/2000 (H1N2) H1N2/2000, A/Damme/IDT5673/2006 (H3N2) H3N2/2006, and A/Herford/IDT5932/2007 (H3N2) H3N2/2007. Similar to the A/Bissendorf//IDT1864/2003 strain, the strains (H1N1/1981, H1N2/2000 and H3N2/2007) analyzed here, were also able to infect ciliated cells and mucus-producing cells (Figure 1). Strain H1N1/2006 differed from the other strains; its infection was less efficient as indicated by a lower number of cells expressing viral antigen.

**Figure 2:**
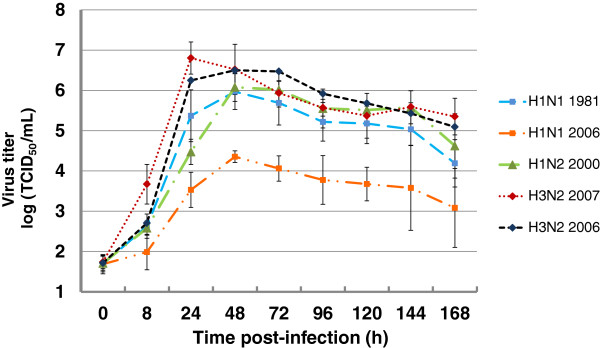
**Infectivity kinetics in supernatants of PCLS infected by swine influenza A viruses.** PCLS were mock-infected or infected by subtypes H1N1, H1N2, H3N2 swine influenza virus. Up to 7 dpi, infectious virus released into the supernatants of PCLS was titrated at daily intervals by endpoint dilution titration (TCID_50_/mL; 50% tissue culture infectious dose/mL).

The infection of well-differentiated respiratory epithelial cells by swFLUAV was analyzed with respect to the kinetics of virus release into the supernatant. PCLS were infected by the five strains mentioned above. The replication efficiency was determined by titration the infectious virus in the supernatant at different time points after infection. As shown in Figure 2, at 24 hours post-infection (hpi) the highest titers were determined for the two H3N2 strains, H3N2/2007 and H3N2/2006, with values ranging between 1 × 10^6^ and 1 × 10^7^ TCID_50_/mL. These values increased or decreased only slightly by 48 hpi (Figure 2). The H1N1 strain H1N1/1981 and the H1N2 strain H1N2/2000 replicated somewhat slower, reaching the maximum titer of 1 × 10^6^ TCID_50_/mL at 48 hpi. Strain H1N1/2006 differed significantly from the other four strains. It multiplied with the same kinetics as the strains H1N1/1981 and H1N2/2000; however, the maximum titer reached at 48 hpi was only 4.35 × 10^4^ TCID_50_/mL which is about 60 to 200- fold lower than those of the other four strains.

**Figure 3:**
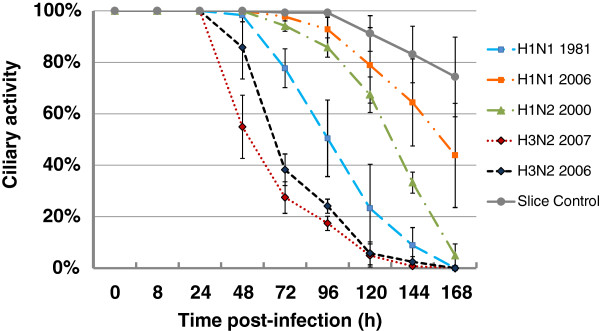
**Ciliary activity of swine PCLS infected by swine influenza A viruses.** PCLS were mock-infected or infected by subtypes H1N1, H1N2, H3N2 swine influenza virus. Up to seven dpi, PCLS were analyzed for ciliary activity at daily intervals.

The five strains were also compared for their ability to induce a ciliostatic effect. As shown in Figure 3, in this respect the two H3N2 strains were found to be most virulent. In PCLS infected by strains H3N2/2007 and H3N2/2006, about 50% of the epithelial cells had lost ciliary activity between 48 and 72 hpi; by about 108 hpi, ciliary activity was detectable only on about 10% of the airway epithelial cells. The H1N1 strain H1N1/1981 and the H1N2 strain H1N2/2000 were somewhat less virulent requiring about 96 h and 132 h, respectively, to reach the 50% value, and 144 h and 160 h to reach the 10% value. The lowest ciliostatic effect was found with the H1N1 strain H1N1/2006. In PCLS infected by this strain, 50% of the airway epithelium had retained ciliary activity even seven dpi. The actual percentage of the epithelium that retained ciliary activity is even higher if one takes into account that in the control sample only 75% of the luminal epithelium showed ciliary activity seven dpi. This reduction is due to the fact that the medium was not changed during the time course of infection. No loss of ciliary activity is observed if the medium is changed at daily intervals. Medium change was avoided in the experiments related to Figure 3, to have the same conditions as in the virus growth experiment shown in Figure 2.

The virulence of swFLUAV strains H1N1/1981, H1N1/2006, H1N2/2000, and H3N2 2006 was analyzed in experimental infection of animals by determining the dyspnoea score, the rectal temperature, and the viral load in the lung. A trial with the second H3N2 strain (A/sw/Herford/IDT5932/2007) was not performed because both H3N2 viruses provided similar results in the in vitro investigations. Coughing was only observed rarely and inconsistently and therefore not analyzed.

**Figure 4:**
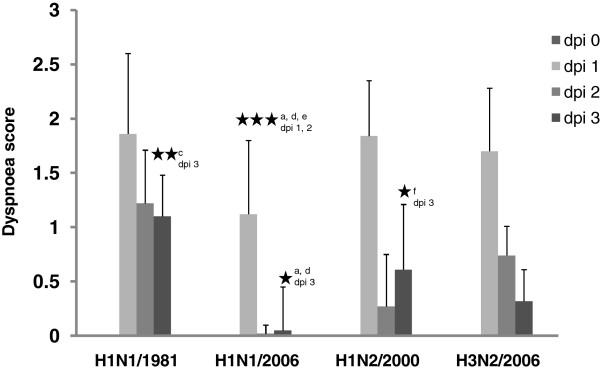
**Dispnoea score.** Dyspnoea (score) observed in pigs after experimental infection with influenza A viruses, dpi, days post infection; statistics: asterisk (1 *p* < 0.05, 2 *p* < 0.010, 3 *p* < 0.005, comparison of following groups: a H1N1/1981:H1N1/2006, b H1N1/1981:H1N2/2000, c H1N1/1981:H3N2/2006, d H1N1/2006:H1N2/2000, e H1N1/2006:H3N2/2006, f H1N2/2000:H3N2/2006).

With respect to the dyspnoea score, the H1N1/1981 strain was most virulent showing high values even three days after infection (Figure 4). Only low virulence was determined for the strain H1N1/2006 which showed increased values only on day 1 pi, and even this value was significantly lower (*p* < 0.001) than those determined for the other three viruses. Strains H1N2/2000, and H3N2/2006 were intermediate; while their dyspnoea scores on day 1 pi were similar to those of H1N1/1981, these values dropped significantly faster on the following two days than those of H1N1/1981 (*p* < 0.05).

**Figure 5:**
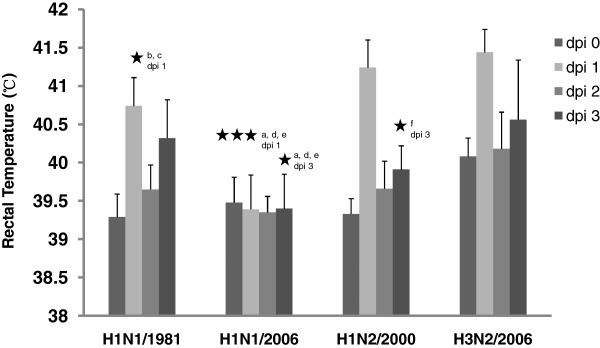
**Rectal temperature.** Rectal temperatures (°C) in pigs after experimental aerosol infection with swine influenza A viruses, dpi, days post infection; statistics: asterisk (1 *p* < 0.05, 2 *p* < 0.010, 3 *p* < 0.005, comparison of following groups: a H1N1/1981:H1N1/2006, b H1N1/1981:H1N2/2000, c H1N1/1981:H3N2/2006, d H1N1/2006:H1N2/2000, e H1N1/2006:H3N2/2006, f H1N2/2000:H3N2/2006).

Infection of pigs with strain H1N1/2006 did not affect the rectal temperature (Figure 5). Increased temperature values were determined for the other three viruses. As far as the change in the rectal temperature is concerned, strain H3N2/2006 was able to induce stronger fever reactions which is supported by a significant difference to strain H1N1/1981 on day 1 pi (*p* < 0.001) and to strain H1N2/2000 on day 3 pi (*p* < 0.05), respectively.

**Figure 6:**
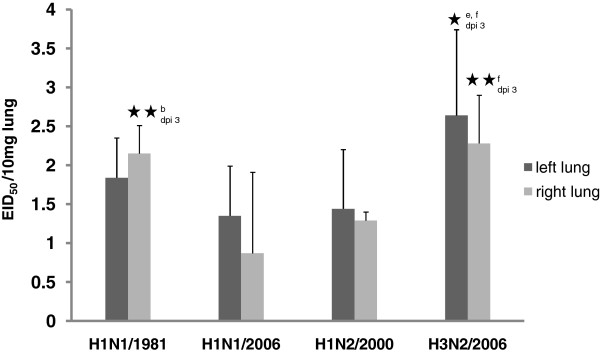
**Viral lung load.** Viral lung load (EID_50_/10 mg lung) in pigs 3 days after aerosol infection with swine influenza A viruses; statistics: asterisk (1 *p* < 0.05, 2 *p* < 0.010, 3 *p* < 0.005, comparison of following groups: a H1N1/1981:H1N1/2006, b H1N1/1981:H1N2/2000, c H1N1/1981:H3N2/2006, d H1N1/2006:H1N2/2000, e H1N1/2006:H3N2/2006, f H1N2/2000:H3N2/2006).

The viral load was determined for both, the right and the left half of the lung (Figure 6). In general, the highest infectivity values were measured for strain H3N2/2006 followed by strain H1N1/1981. Due to the individual variation, significant differences were determined only in some cases, e.g. significantly increased values for the H3N2 in the left lung compared to H1N1/2006 (H1N1) and H1N2/2000 (*p* < 0.05).

## Discussion

Our analysis of the virulence of swFLUAVs shows that the three strains H3N2/2006, H1N1/1981, H1N2/2000 cause disease in pigs as evidenced by a rise in the parameters dyspnoea, rectal temperature, and virus load. Low virulence was determined for strain H1N1/2006 which showed humble dyspnoea values and no increase in rectal temperature. The analysis of the other three strains shows that disease symptoms may vary as indicated by the finding that changes in one parameter were most prominent for one strain, whereas another strain was affected more at a different parameter. The dyspnoea values decreased in the order H1N1/1981 > H1N2/2000 / H3N2/2006 > H1N1/2006. The rectal temperature decreased in the order H3N2/2006 > H1N2/2000 > H1N1/1981 > H1N1/2006. In the viral lung load, the order was H3N2/2006 / H1N1/1981 > H1N2/2000 > H1N1/2006. Despite these differences there was one remarkable common feature with respect to all parameters: the low virulence of H1N1/2006. Interestingly, strain H1N1/2006 was isolated from a diseased animal. Similar to other current European H1N1 swFLUAV strains, it is characterized by a low virulence in animal experiments [30]. By contrast, when the avian-like H1N1 viruses were introduced in the European swine populations in 1979 [4], they were characterized by a pronounced pathogenicity similar to that of strain H1N1/1981 [31]. Over the years, viruses of this subtype appear to have adapted to pigs in such a way that they are still transmitted successfully but only develop mild disease symptoms in animal infection experiments. The fact that they still can cause disease in pig populations is an interesting observation and may be explained by the influence of additional factors for example by the effect of bacterial co-infections [32].

PCLS are a promising culture system to analyze the infection of differentiated respiratory epithelial cells. From our data we suggest that the characteristics of infection determined here for four swFLUAV strains reflect the pathogenic properties of these viruses. The strain with a low pathogenicity, A/Bad Griesbach/IDT5604/2006 (H1N1), differed from the other strains not only in a significantly lower amount of virus released into the supernatant but also in a lower ciliostatic effect. It will be interesting in the future to find out whether complete ciliostasis is an indicator for the virulence of swFLUAV, whereas low virulent swFLUAVs may be able only to cause partial ciliostasis. Such a marker would greatly facilitate the screening of treatments or antivirus substances [31,33] for their effect on pathogenicity and thus reduce the number of animal experiments.

PCLS may also help to distinguish between parameters of virulence. The H3N2 strains were superior to H1N1 and H1N2 strains in both virus release and ciliostatic effect. Among virulence parameters in infected pigs, the H3N2 virus showed the largest effect on rectal temperature and virus load and reflected also a high dyspnoea score. Though more experiments are required to confirm these suggestions, the value of an in vitro-system that predicts virulence is worth the effort.

## Competing interests

The authors declare that they have no competing interests.

## Authors’ contributions

FM, DP, IHP, CSW, XR, and GH conceived and designed the experiments; FM, DP, and SU performed the experiments; FM, IHP, CSW, XR, and GH analyzed the data; RD designed, performed and analyzed the infection trials in pigs; IHP and RD contributed reagents/materials/analysis tools; FM, GH, RD and XR wrote or helped to draft the paper. All authors read and approved the final manuscript.
